# Population structure analysis of the neglected parasite *Thelazia callipaeda* revealed high genetic diversity in Eastern Asia isolates

**DOI:** 10.1371/journal.pntd.0006165

**Published:** 2018-01-11

**Authors:** Xi Zhang, Ya Li Shi, Lu Lu Han, Chen Xiong, Shi Qi Yi, Peng Jiang, Zeng Xian Wang, Ji Long Shen, Jing Cui, Zhong Quan Wang

**Affiliations:** 1 Department of Parasitology, School of Basic Medical Sciences, Zhengzhou University, Zhengzhou, China; 2 Department of Microbiology and Parasitology, Anhui Medical University, Anhui, China; Negrar Hospital, ITALY

## Abstract

**Background:**

*Thelazia callipaeda* is the causative agent of thelaziasis in canids, felids and humans. However, the population genetic structure regarding this parasite remains unclear.

**Methodology/principal findings:**

In this study, we first explored the genetic variation of 32 *T*. *callipaeda* clinical isolates using the following multi-molecular markers: *cox*1, *cyt*b, 12S rDNA, ITS1 and 18S rDNA. The isolates were collected from 13 patients from 11 geographical locations in China. Next, the population structure of *T*. *callipaeda* from Europe and other Asian countries was analyzed using the *cox*1 sequences collected during this study and from the GenBank database. In general, the Chinese clinical isolates of *T*. *callipaeda* expressed high genetic diversity. Based on the *cox*1 gene, a total of 21 haplotypes were identified. One only circulated in European countries (Hap1), while the other 20 haplotypes were dispersed in Korea, Japan and China. There were five nucleotide positions in the *cox*1 sequences that were confirmed as invariable among individuals from Europe and Asia, but the sequences were distinct between these two regions. Population differences between Europe and Asian countries were greater than those among China, Korea and Japan. The *T*. *callipaeda* populations from Europe and Asia should be divided into two separate sub-populations. These two groups started to diverge during the middle Pleistocene. Neutrality tests, mismatch distribution and Bayesian skyline plot (BSP) analysis all rejected possible population expansion of *T*. *callipaeda*.

**Conclusions:**

The Asian population of *T*. *callipaeda* has a high level of genetic diversity, but further studies should be performed to explore the biology, ecology and epidemiology of *T*. *callipaeda*.

## Author summary

*Thelazia callipaeda* is the causative agent of thelaziasis canids, felids and humans. Despite the existing threat of thelaziosis in China, the genetic diversity of *T*. *callipaeda* has not been investigated across its wide geographical distribution in China, yet such information may provide insight into the disease epidemiology and the development of specific control measures. In this study, the genetic variation of 32 *T*. *callipaeda* clinical isolates collected from 13 patients from 11 geographical locations in China were explored using the following multi-molecular markers: *cox*1, *cyt*b, 12S rDNA, ITS1 and 18S rDNA. In addition, the population structure of *T*. *callipaeda* from Europe and other Asian countries was analyzed using the *cox*1 sequences collected during this study and from the GenBank database. In general, the Chinese clinical isolates of *T*. *callipaeda* demonstrated high genetic diversity. Based on the *cox*1 gene, a total of 21 haplotypes were identified, one circulated in European countries (Hap1), while the other 20 haplotypes were dispersed in Korea, Japan and China. There were five nucleotide positions in the *cox*1 that were confirmed as invariable among individuals from Europe and Asia, but the sequences were distinct between these two regions. Population differences between Europe and Asian countries were greater than those among China, Korea and Japan, such that the *T*. *callipaeda* population from Europe and Asia should be divided into two separate sub-populations. These two groups started to diverge during the middle Pleistocene.

## Introduction

The spirurid nematode *Thelazia callipaeda* (Spirurida: Thelaziidae) is the major etiological agent of ocular thelaziosis [1]. This worm can parasitize the conjunctival sac of domestic and wild carnivores and humans, causing conjunctivitis, lacrimation and itchiness, and even blindness [2,3]. The parasite is transmitted by a drosophilid insect of the genus *Phortica* (Diptera, Drosophilidae), that feeds on the lachrymal secretions of mammals [4,5]. *T*. *callipaeda* was previously known as the oriental eyeworm because of its original description in eastern Asia countries [1]. Since the first cases of canine thelaziosis reported in Italy in 1988, the nematode has spread through many southern, central, western and eastern European countries [6]. A broad spectrum of wild carnivores, such as wolves, wildcats, red foxes, badgers, beech martens and even brown hares, plays an important role in maintaining and spreading eyeworm infections to domestic animals and humans [7]. The first human case was described in Beijing, China in 1917 [8]. Although sporadic human thelaziosis has been reported in several European countries, human infections are mainly documented in people living in China, Japan, Korea and India [9]. China is most likely to have the largest number of cases of thelaziasis in the world with more than 600 cases reported to date [10]. Recently, an increase in *T*. *callipaeda* infections has been reported in animals and humans living in European countries and in China [6,11].

Consequently, *T*. *callipaeda* poses a serious threat to public health and thelaziosis has even been termed as an emerging enzootic disease [1,6,9]. Understanding the host specificity, transmission pattern and population genetic characteristics of the parasite are valuable for the prevention and control of thelaziasis in animals and humans [12]. However, our knowledge regarding these issues is still fragmented, and insufficient studies on population genetics of *T*. *callipaeda* have been carried out. One possible reason is that as a neglected pathogen, *T*. *callipaeda* does not draw enough attention from most parasitologists. This may also be attributed to the difficulty in collecting *T*. *callipaeda* isolates from different hosts and distinct geographical locations.

More than 10 years ago, Otranto and Traversa [13] performed the first genetic variance analysis among *Thelazia* species by using the first internal transcribed spacer (ITS1) ribosomal DNA sequence, and concluded that the ITS1 sequence was a useful genetic marker for the molecular identification of *Thelazia* spp. In 2005, Otranto et al. [12] investigated the genetic variability among 50 individual adult specimens of *T*. *callipaeda* from Europe and Asia based on the mitochondrial cytochrome *c* oxidase subunit 1 gene (*cox*1). Recently, although various publications of the *T*. *callipaeda* infections in animals and humans have been reported [3,14–18], no subsequent related studies about its population genetics have been reported. In this study, we explored the genetic variability within human *T*. *callipaeda* isolates collected from different geographical locations in China by using three mitochondrial genes and two nuclear ribosomal DNA sequences as follows: mitochondrial cytochrome *b* (*cyt*b), the small subunit of ribosomal DNA gene (12S rDNA) and *cox*1; ITS1 and the small subunit of nuclear ribosomal DNA (18S rDNA). These molecular markers were selected because they are suitable for inferring population differences and conducting phylogenetic analysis at different taxonomic levels [12,13,19–21]. Using *cox*1, we also performed a genetic variability comparative analysis on clinical *T*. *callipaeda* isolates collected in China and from previous publications. Additionally, the presumed transmission pattern of *T*. *callipaeda* investigated here relied on phylogeny and molecular dating methods.

## Materials and methods

### Ethics statement

This study was approved by the Life Science Ethics Committee of Zhengzhou University (No. 2017–0006). The protocol and written informed consent form were approved by the Human Ethics Committees of the Zhengzhou University. All subjects older than eighteen years old provided written informed consent; in the case of children, they provided written informed assent, and their parents/guardians provided written consent for them. All worms were collected from patients to treat their thelaziasis and not expressly for the purpose of the present study.

### Parasite collection and identification

A total of 32 worms were harvested from 13 patients from 11 distinct geographical locations in China from September 2007 to July 2016 (Table 1). All nematodes were removed from the eyes of patients with intraocular forceps, while the patients were anesthetized with oxybuprocaine. The collected nematodes were transferred to Petri dishes containing physiological saline (0.9% NaCl). These eyeworms were identified as *T*. *callipaeda* according to the morphological characteristics (e.g., shape of the buccal capsule, presence of transversally striated cuticle and cloacal papillae, morphology of the spicules in males and the position of the vulva in females) described in Otranto et al. [22].

**Table 1:** Geographical and host origins of *Thelazia callipaeda* specimens studied herein.

Geographical origin	Host origin	SS^a^	Haplotypes^b^	References
Italy (Piemonte)	*Canis familiaris*	5	h1	Otranto et al. (2005)
Italy (Piemonte)	*Vulpes vulpes*	2	h1	Otranto et al. (2005)
Italy (Basilicata)	*Felis catus*	5	h1	Otranto et al. (2005)
Italy (Basilicata)	*C*. *familiaris*	11	h1	Otranto et al. (2005)
Italy (Basilicata)	*V*. *vulpes*	2	h1	Otranto et al. (2005)
Italy (Calabria)	*C*. *familiaris*	5	h1	Otranto et al. (2005)
Germany	*C*. *familiaris*	6	h1	Otranto et al. (2005)
The Netherlands	*C*. *familiaris*	1	h1	Otranto et al. (2005)
Portugal (Vinhais)	*Oryctolagus cuniculus*	4	h1	Gama et al. (2016)
Serbia (Baljevac)	*C*. *familiaris*	1	h1	Gajic et al. (2014)^c^
Serbia (Baljevac)	*F*. *catus*	1	h1	Gajic et al. (2014)^c^
Romania (Oradea, Bihor)	*C*. *familiaris*	3	h1	Mihalca et al. (2015)
Romania	*C*. *familiaris*	2	h1	Ionita et al. (2015)^c^
Slovakia	*C*. *familiaris*	1	h1	Cabanova (2017)^c^
Hungary	*F*. *catus*	1	h1	Takacs and Farkas (2016)^c^
China (Anhui)	*C*. *familiaris*	5	h2 (1), h3 (1), h4 (2), h5 (1)	Otranto et al. (2005)
Korea (Kou Kuk)	*C*. *familiaris*	8	h5 (1), h6 (1), h7 (2), h8 (4)	Otranto et al. (2005)
Japan (Okayama)	*Homo sapiens*	9	h9	Kumase et al (2009)^c^
Japan (Saitama)	*C*. *familiaris*	1	h10	Yoshikawa (2013)^c^
Japan (Tokyo)	*C*. *familiaris*	7	h10 (5), h11 (1), h12 (1)	Yoshikawa (2013)^c^
China (Anhui, Hefei)	*H*. *sapiens*	1	h13	this study
China (Anhui, Liuan)	*H*. *sapiens*	6	h13	this study
China (Liaoning, Dandong)	*H*. *sapiens*	2	h14	this study
China (Shaanxi, Shangluo)	*H*. *sapiens*	3	h15	this study
China (Shaanxi, Tongchuan)	*H*. *sapiens*	3	h16	this study
China (Hubei, Huanggang)	*H*. *sapiens*	2	h17	this study
China (Hubei, Wuhan)	*H*. *sapiens*	1	h18	this study
China (Henan, Pingdignshan)	*H*. *sapiens*	8	h15 (4), h19 (4)	this study
China (Henan, Jiaozuo)	*H*. *sapiens*	2	h20	this study
China (Henan, Luoyang)	*H*. *sapiens*	2	h15	this study
China (Henan, Zhengzhou)	*H*. *sapiens*	2	h15 (1), h21 (1)	this study

a, sampling size

b, haplotypes identified based on the *cox*1

c, direct submitted sequences.

### Sequencing of target genes and alignment

Total genomic DNA was extracted from individual specimens using the EasyPure Genomic DNA Kit (Transgen, China) following the manufacturer’s protocol. Five molecular markers, viz. *cyt*b, *cox*1, 12S rDNA, ITS1 and 18S rDNA, were amplified to explore the genetic diversity of *T*. *callipaeda*. For 12S, ITS1 and *cox*1, the amplifications were obtained using primer combinations described in Casiraghi et al. (2004) [23], Otranto and Traversa (2004) [13], and Otranto et al. (2005) [12], respectively. The forward and reverse primers used for amplifying the *cyt*b and 18S markers were designed as follows: cobF 5′-TGATTGGTGGTTTTGGTAA-3′; and cobR 5′- ATAAGTACGAGTATCAATATC-3′; and 18SF 5′- CTCATAAAATAATTGG TGAATCTGAATAGC-3′ and 18SR 5′-ATAACTTTTCAGCAATGGTTACAG-3′, respectively. PCR products were purified using the EasyPure PCR Purification Kit (Transgen, China) and sequenced in both directions at the Genwiz Company (Beijing, China). All sequences were deposited in the GenBank database (S1 Table). The sequenced genes were initially aligned using the default settings in the program Clustal X v.2.0 [24] and adjusted in MEGA v.6.06 [25] according to the corresponding amino acid sequences of protein-coding genes and secondary structure of ribosomal DNA sequences. The nucleotide composition, conserved sites, variable sites, parsimony-informative sites, and singleton sites were estimated using MEGA v.6.06.

### Genetic diversity analysis of *T*. *callipaeda* isolates from China

The program DnaSP v5.10 [26] was employed to analyze the number of haplotypes, haplotype diversity (Hd), and nucleotide diversity (Pi) of each molecular marker. Network v5.0 [27] was used to draw a median-joining network to analyze the relationships among the detected haplotypes. Analysis of molecular variance (AMOVA) was computed in Arlequin v.3.5 [28] with non-parametric permutations of 1,000 times (*p* = 0.05) to detect the partitions of genetic diversity within and among populations. Pairwise *F*_ST_ values between populations were performed for all datasets in Arlequin to explore levels of genetic differentiation among the populations. The significance of *F*_ST_ values evaluated was based on 1000 random permutations. Demographic changes were also estimated using mismatch distributions in Arlequin with 1000 simulations, under a scenario of no recombination. The validity of the expansion model was tested by using the sum of squared deviations (SSD) and raggedness index (RI) between observed and expected mismatches. The neutrality tests using Tajima’s *D* [29] and Fu’s *F*_S_ [30] were also applied through Arlequin as an assessment of possible population expansion.

### Genetic variability comparative analysis of *T*. *callipaeda* isolates using *cox*1

To make a worldwide genetic variability comparative analysis of *T*. *callipaeda*, all available sequences of *cox*1 in the GenBank database were included (S1 Table). In addition, we performed a Bayesian skyline plot analysis (BSP) implemented in BEAST v1.8.2 [31] to estimate the change in population size over time, and the time to the most recent common ancestor (tMRCA) for each *T*. *callipaeda* haplotype. A piecewise-constant skyline model was selected. The molecular evolutionary rate of *cox*1 was fixed at 0.01 substitutions per site per million years ago (Mya) according to the substitution rate for nematode mtDNA [32]. Tracer v1.5 [33] was used to reconstruct the demographic history over time.

### Phylogenetic diversity analysis

The phylogenetic pattern of all *cox*1 haplotypes was estimated through maximum parsimony (MP) and Bayesian inference (BI). MP analysis was performed in MEGA v.6.06. Confidence in each node was assessed by boot-strapping (1000 pseudo-replicates). Bayesian inference was performed in MrBayes v.3.2 [34], after determining the appropriate substitution model by applying the Akaike information criterion (AIC) in jModelTest 2 [35]. The analysis consisted of two runs, each with four MCMC chains running for 5, 000, 000 generations, and sampling every 100th generation. Stationarity was assessed using a convergence diagnostic. An average standard deviation of the split frequencies (ASDSF) < 0.01 was used as criteria for convergence between both runs. The consensus tree was drawn after removing the first 10 000 trees (20%) as the burn-in phase. The approximate divergence time was estimated using an uncorrelated log-normal relaxed molecular-clock model in the BEAST v1.8.2 program. The substitution model was assigned following model selection by jModelTest 2. For the earlier tree, a basic coalescent model was chosen, assuming a constant population size over the given time period considered. Two replicate MCMC runs were performed, with the tree and parameter values sampled every 1, 000 steps over a total of 1×10^8^ steps.

## Results

### Genetic diversity of Chinese *T*. *callipaeda* isolates

**Fig 1:**
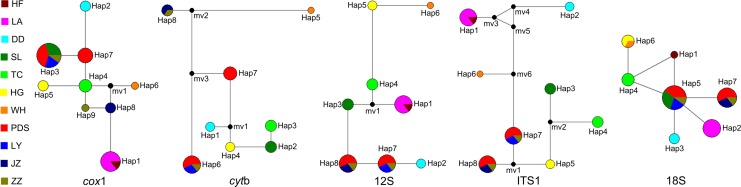
*Thelazia callipaeda* haplotype networks by using *cox*1, *cyt*b, 12S rDNA, ITS1 and 18S rDNA. Each haplotype is represented by a circle, with the area of the circle proportional to its frequency. The median vector is indicated by a solid black circle. Geographic regions are designated as follows for China: Hefei (HF), Liuan (LA), Dandong (DD), Shangluo (SL), Tongchuan (TC), Huanggang (HG), Wuhan (WH), Pingdingshan (PDS), Luoyang (LY), Jiaozuo (JZ) and Zhengzhou (ZZ).

The sequence alignments for *cyt*b, *cox*1, 12S, ITS1 and 18S were 1035 bp, 660 bp, 453 bp, 772 bp and 1201 bp, respectively. In 32 isolates from eleven localities, the genetic markers of *cox*1, 12S, ITS1 and 18S were used to identify 9, 8, 8 and 7 new haplotypes (Haps),. For *cyt*b, 8 Haps were identified in only 25 isolates because the sequences of isolates from Hefei (HF) and Liuan (LA) of Anhui province were not amplified (S2 Table). With the exception of isolates from Pingdingshan (PDS) and Zhengzhou (ZZ) of Henan province, which shared two haplotypes, each geographic population shared only a single haplotype. Analyses of the median-joining networks (Fig 1) showed that samples from HF and LA shared a haplotype (Hap1) when using *cox*1, 12S and ITS1, but under the analysis with 18S, these samples identified two Haps (Hap1 and Hap2). Clinical eyeworms from Dandong (DD) and Tongchuan (TC) shared geographical specific Haps using each of the selected markers. Isolates from Huanggang (HG) and Wuhan (WH) also revealed specific Haps when using *cyt*b, *cox*1, 12S and ITS1; however, these samples shared a single haplotype using 18S. Using *cyt*b, 12S and ITS1, a single geographical specific haplotype was identified in *T*. *callipaeda* from Shangluo (SL). Interestingly, using *cox*1 (Hap3) and 18S (Hap5), these isolates from SL shared the same haplotype with all samples from Luoyang (LY), 4 samples from Pingdingshan (PDS) and one from Zhengzhou (ZZ). The remaining isolates from PDS and ZZ shared the same haplotype using 12S, ITS1 and 18S. However, when using *cyt*b and *cox*1, the remaining isolates possessed two distinct Haps. Using *cyt*b, 12S, ITS1 and 18S, worms from Jiaozuo (JZ) shared the same haplotype with the second sample from ZZ. Only with *cox*1, did the JZ isolates identify as a distinct haplotype. The results from the analysis of molecular variance (AMOVA) showed that much more genetic variance lay among the populations than within the populations for all datasets, more specifically, *cyt*b: 69.77% *vs*. 30.23%; *cox*1: 91.32% *vs*. 8.68%; 12S: 94.26% *vs*. 5.74%; ITS1: 91.69% *vs*. 8.31%; 18S: 83.71% *vs*. 16.29% (Table 2). The pairwise fixation index (*F*_ST_) values between specified geographical regions were estimated for all molecular markers used to measure the population differentiation (S3 Table). With the exception of *F*_ST_ values between PDS and the remaining populations, and ZZ and the remaining populations, most of the *F*_ST_ values reached 1.00. No estimated pairwise *F*_ST_ values were statistically significant besides those between LA and the remaining populations, and PDS and the remaining populations. Based on all genes, the neutrality tests of Tajima's *D* and Fu's *F*_S_ for the total population showed non-significant positive values, except for the negative value of Fu's *F*_S_ (-1.65282, *p* = 0.164), using the 18S gene (Table 3). Using all markers, mismatch distribution analyses revealed multi-modal frequency distributions for the total population, rejecting possible population expansion (S1 Fig). In addition, low values were found for the sum of squared deviation (SSD) and raggedness index (RI) under the demographic expansion model (Table 3).

**Table 2:** Analysis of molecular variance (AMOVA) of the populations of *Thelazia callipaeda* from China.

Markers	Source of variation							
	Among populations				Within populations			
	d.f	Sum of squares	Variance components	Percentage of variation	d.f	Sum of squares	Variance components	Percentage of variation
*cyt*b	8	105.360	4.32759	69.77%	16	30.000	1.87500	30.23%
*cox*1	10	57.250	2.00345	91.32%	21	4.000	0.19048	8.68%
12S	10	55.156	1.95351	94.26%	21	2.500	0.11905	5.74%
ITS1	10	112.406	3.93972	91.69%	21	7.500	0.35714	8.31%
18S	10	18.094	0.61188	83.71%	21	2.500	0.11905	16.29%

d.f = degrees of freedom.

**Table 3:** Mismatch and neutrality test results of the *Thelazia callipaeda* population from China.

Markers	Neutrality Tests		Mismatch	
	Fu’s *F*_S_	Tajima’s *D*	SSD	RI
cytb	5.59212 (0.974)	0.95309 (0.864)	0.02703 (0.432)	0.05787 (0.403)
cox1	0.44453 (0.596)	1.04997 (0.891)	0.02243 (0.409)	0.07200 (0.433)
12S	0.97235 (0.707)	0.79949 (0.815)	0.02074 (0.459)	0.03965 (0.716)
ITS1	4.53458 (0.934)	1.91959 (0.974)	0.04715 (0.021)	0.11855 (0.044)
18S	-1.65282 (0.164)	0.18826 (0.618)	0.01384 (0.056)	0.12259 (0.068)

SSD = Sum of squared deviation, RI = Raggedness index. Number in parentheses is the *P* value.

### Compare the genetic variability between Chinese isolates and isolates from European and other Eastern Asia countries

A total 21 haplotypes were identified within *T*. *callipaeda* isolates from 25 localities in 12 countries (Table 1). Hap1 was only found in *T*. *callipaeda* from European countries (Italy, Germany, The Netherlands, Portugal, Serbia, Romania, Slovakia, Hungary, etc). The remaining haplotypes were shared by samples from Eastern Asia countries (Korea, Japan and China). Haps2–8 and haps10–12 were shared by samples isolated from domestic dogs (*Canis familiaris*). Hap9 and haps13–21 were identified in eyeworms collected from humans (*Homo sapiens*). The alignment of 21 haplotypes revealed nucleotide variations (36 transitions and 1 transversion) at 37 alignment positions (S2 Fig). The majority of the nucleotide variability was at the third codon position (n = 31; 83.8%), whereas the remainder was at the first codon position (n = 6; 16.2%). Of the 37 variable positions, only two sites generated nonsynonymous mutations. One was at alignment position 189 (A—G) of Hap4, which changed the amino acid from Methionine to Valine. The other, also located in Hap4 (position 252: C—T), changed Leucine to Phenylalanine. Five nucleotides (i.e., G—A at alignment positions 89, 149, 206 and 257; and C—T at position 539) were invariable among all individuals from Europe and among all individuals from Eastern Asia (Korea, Japan and China), but they were different between Europe and Asia. The pairwise comparisons among 21 haplotypes ranged from 0.15 to 2.86% (Table 4). Within each country studied, the intraspecific divergences in 4 haplotypes from Korea, 4 haplotypes from Japan and 13 haplotypes from China were 0.31–1.09, 0.15–2.55 and 0.15–1.73%, respectively. The AMOVA results showed that much more genetic variance lay among the populations (74.79%) than within the populations (25.21%). Between geographical regions, the population differences (*F*_ST_ value) were 0.961 for Europe *vs*. Korea, 0.866 for Europe *vs*. Japan, 0.827 for Europe *vs*. China, 0.444 for Korea *vs*. Japan, 0.275 for Korea *vs*. China, and 0.430 for Japan *vs*. China (S4 Table).

**Table 4:** Pairwise comparison of sequence differences (%) among the 21 *cox*1 haplotypes, representing *Thelazia callipaeda* from different geographical origins.

Hap	h1	h2	h3	h4	h5	h6	h7	h8	h9	h10	h11	h12	h13	h14	h15	h16	h17	h18	h19	h20	h21
h1	–																				
h2	1.73	–																			
h3	1.57	0.47	–																		
h4	1.25	1.09	0.94	–																	
h5	2.06	0.94	0.78	1.42	–																
h6	1.89	0.78	0.62	1.25	1.10	–															
h7	1.57	0.47	0.31	0.94	0.78	0.62	–														
h8	1.89	0.78	0.62	1.25	1.10	0.31	0.62	–													
h9	2.54	1.73	1.57	1.89	2.06	1.89	1.57	1.89	–												
h10	1.90	1.10	0.94	1.57	0.47	1.26	0.94	1.26	2.22	–											
h11	2.70	1.89	1.73	2.05	2.22	2.05	1.73	2.05	0.15	2.38	–										
h12	2.86	2.05	1.89	2.21	2.38	2.21	1.89	2.21	0.31	2.54	0.15	–									
h13	1.41	0.62	0.78	0.78	1.25	1.09	0.78	1.09	1.73	1.41	1.89	2.05	–								
h14	2.38	1.26	1.10	1.73	0.94	0.78	1.10	0.78	2.38	0.78	2.54	2.71	1.57	–							
h15	2.06	0.94	0.78	1.42	0.62	1.10	0.78	1.10	2.06	0.47	2.22	2.38	1.25	0.62	–						
h16	1.57	0.47	0.31	0.94	0.47	0.62	0.31	0.62	1.57	0.62	1.73	1.89	0.78	0.78	0.47	–					
h17	1.57	0.78	0.62	0.94	0.78	0.94	0.62	0.94	1.89	0.94	2.06	2.22	0.78	1.09	0.47	0.31	–				
h18	1.57	0.47	0.31	0.94	0.78	0.62	0.31	0.62	1.57	0.94	1.41	1.57	0.78	1.10	0.78	0.31	0.62	–			
h19	1.89	0.78	0.62	1.26	0.47	0.94	0.62	0.94	1.89	0.31	2.06	2.22	1.09	0.47	0.15	0.31	0.62	0.62	–		
h20	1.57	0.47	0.31	0.94	0.78	0.62	0.31	0.62	1.57	0.94	1.73	1.89	0.47	1.10	0.78	0.31	0.62	0.31	0.62	–	
h21	1.73	0.62	0.47	1.10	0.62	0.78	0.47	0.78	1.73	0.78	1.89	2.05	0.62	0.94	0.62	0.15	0.47	0.47	0.47	0.15	–

### Phylogenetic diversity and demographic estimation

**Fig 2:**
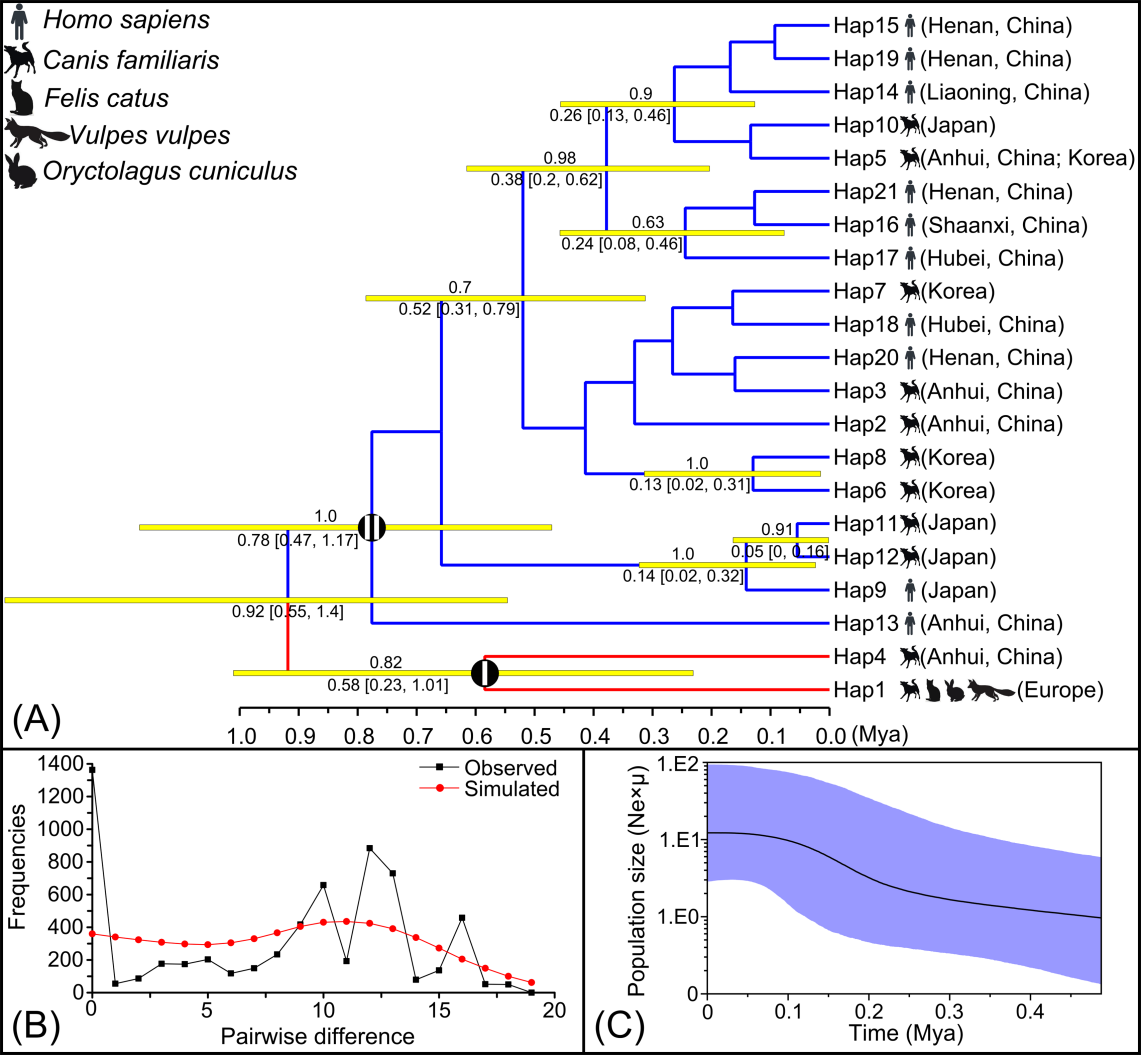
(A) Phylogram of *Thelazia callipaeda* isolates from Europe and Asia with divergence time estimation. Yellow bars at each node show the 95% highest posterior density interval for the main nodes. Numbers above branches represent the Bayesian posterior probabilities. Only posterior probabilities above 0.6 are shown. Numbers below branches indicate the estimated age and 95% confidence intervals (shown in square brackets). (B) Mismatch distribution analysis for the *T*. *callipaeda* population. The line charts represent the observed frequencies of pairwise differences among haplotypes. (C) Bayesian skyline plot calculates for the *T*. *callipaeda* population. The X-axis is in units of million years ago (Mya) and the Y-axis is Ne×μ (effective population size × mutation rate per site per generation). The median estimates are shown as thick solid lines, and the 95% HPD limits are shown by the colored areas.

The likelihood models identified by the jModelTest (AIC) suggested that the HKY+G model was most suitable for *cox*1 haplotypes. Both maximum parsimony and Bayesian analyses generated consistent tree topologies (Fig 2A and S3 Fig). Among the tested haplotypes, Hap1 and Hap4 composed a single clade (clade I), and the remaining haplotypes made up another clade (clade II). Within clade II, the earliest diversifications gave rise to Hap13, then to Hap9, Hap11 and Hap12 (Japanese haplotypes without Hap10). The next diversification event separated the remaining haplotypes. The molecular dating analysis suggested that the two clades began to diverge during the middle Pleistocene (Fig 2A). The time of origin of clade I is estimated to be approximately 0.58 Mya (late Pleistocene) with a 95% highest posterior density (HPD) of 0.23–1.01 Mya. Clade II started to develop in the middle Pleistocene (0.78 Mya) with a 95% HPD of 0.47–1.17 Mya. The early branching of the Japanese haplotypes (without Hap10) started in the late Pleistocene (0.14 Mya, with a 95% HPD of 0.02–0.32 Mya). Neutrality tests of Tajima's *D* and Fu's *F*_S_ for the total population showed non-significant positive values, thereby rejecting possible population expansion (S5 Table). Mismatch distribution analyses revealed multi-modal frequency distributions (Fig 2B). In addition, low values for the sum of squared deviation and raggedness index under the demographic expansion model were found (S5 Table). The result of Bayesian skyline plot analysis of the *T*. *callipaeda* population revealed a gradual expansion trend, but also rejected sudden population expansion (Fig 2C).

## Discussion

Although more than 10 species of the spirurid genus *Thelazia* can cause veterinary or medical problems in many parts of the world [13], only *T*. *callipaeda* affects humans in China, causing mild to severe clinical symptom [36]. It is still unknown whether there are other human infective species or genotypes of *T*. *callipaeda* in China. In this study, we collected 32 eye worms from 13 patients from 11 distinct geographical locations in China over a period of 10 years, and performed a genetic variation analysis of these isolates using a multi-gene approach to investigate the population structure of *T*. *callipaeda*.

Haplotypes of the Chinese *T*. *callipaeda* population identified by each molecular marker were generally consistent. Of all the geographic populations, only PDS and ZZ isolates revealed two different haplotypes. Worms from ZZ were harvested in two different patients. However, interestingly, all PDS isolates that were collected from a single child also had two haplotypes, indicating the patient probably experienced two or more infections of drosophilid flies. Each geographic population and the total population demonstrated high haplotype diversity (Hd) values (nearly 1.0) by all markers, however, the nucleotide diversity (Pi) values of each gene were below 0.01, indicating that multiple haplotypes were differentiated by few nucleotide mutations. Correspondingly, the AMOVA results also showed that much more genetic variance exists among the populations than within the populations [19]. The pairwise fixation index (*F*_ST_) was used to measure the population differentiation. Most of the *F*_ST_ values between specified geographical regions were far more than 0.25, indicating very high genetic differentiation and a long-term interruption of gene flow among the geographical populations [37].

Based on the partial *cox*1 sequence, Otranto et al. [12] performed the first genetic variability investigation of the *T*. *callipaeda* population in 2005. Since then, sequences of *T*. *callipaeda* from different geographical locations and different hosts have been published [3, 16–18]. In this study, based on previous research by Otranto et al. [12], we added new data from Chinese clinical isolates and other published data to generate a comprehensive genetic variation analysis of the *T*. *callipaeda* population. Some interesting discoveries were made: (1) Thirteen novel genotypes of *T*. *callipaeda* were identified; four from Japan (Haps9–12), and another nine from Chinese clinical isolates (Haps13–21); within the 4 Japanese genotypes, one was from a human, and the other 3 were from *Canis familiaris*. (2) In *cox*1, Otranto et al. [12] found six nucleotide positions (positions 89, 149, 206, 257, 539 and 608) that were invariable among individuals from Europe and Asia, but they were distinct between Europe and Asia. We confirmed five of them (positions 89, 149, 206, 257, 539 and 608) using an enlarged dataset in this study. (3) Population differences between Europe and Asian countries were larger than those among China, Korea and Japan. Within Asian countries, the genetic difference between China and Korea was the smallest, while the genetic difference between Korea and Japan was the largest. Only within human isolates were the genetic differences among Chinese samples smaller than those between China and Japan. This phenomenon may be attributed to the geographical distance’s influence on gene flow among species [38], and possibly to the geographical origin, which needs to be analyzed further in the future. (4) The *T*. *callipaeda* population from Europe and Asia should be divided into the following two subgroups: one group (clade I) comprising worms from European countries (Hap1) and a sample isolated from a dog in Anhui of China (Hap4); the other group (clade II) comprising *T*. *callipaeda* from Korea, Japan and China. Individuals in clade II diverged earlier than those in clade I. Within the Asian haplotypes, Hap13 (clinical isolates of Anhui) was the ancestral haplotype; then, it was transmitted to Japan, Korea and other regions of China. (5) All of the neutrality tests, mismatch distribution and BSP analyses rejected possible population expansion of *T*. *callipaeda*, indicating this nematode population was in the stable phase [37].

Generally, the population structures of parasites are associated with large genetic differentiation among populations from different geographical regions and/or hosts and low intra-population genetic variability [39,40]. In this study, there was a relatively large genetic differentiation (above 0.82) between the European and Asian populations; however, the genetic variability among Asian countries was small (below 0.44). In addition, phylogenetic analyses supported the conclusion that isolates from Europe and Asia (excluding the Hap4) are separate populations. However, the genetic differentiation of *T*. *callipaeda* from distinct hosts (dogs, foxes, cats and human) was inconspicuous, suggesting a lack of connection between eyeworms and host species [12]. That being said, an important factor to consider is the abundance of genetic diversity in the Asian isolates. A possible explanation for this genetic diversity is that *T*. *callipaeda* populations are tightly linked to the intermediate host (vector). The drosophilid fly *Phortica variegata* was the vector of *T*. *callipaeda* in Europe, whereas the *T*. *callipaeda* in Anhui of China was transmitted by *P*. *okadai* [9,12]. Although it is still unknown whether other vector species exist in Asian countries, considering the high genetic diversity of *T*. *callipaeda* in Korea, Japan and China, it is very likely that some novel species or genotypes of *Phortica* flies exist in these areas. Hence, further studies should be launched to explore the biology, population genetics, ecology and epidemiology of *T*. *callipaeda* in the future.

In summary, the Chinese clinical isolates of *T*. *callipaeda* expressed a high level of genetic diversity. Using the *cox*1 gene, thirteen new genotypes were identified, four from Japan (Haps9–12), and nine from China (Haps13–21). There were five positions in the *cox*1 sequences (89, 149, 206, 257, 539 and 608) that were confirmed invariable among individuals from Europe and Asia, but the sequences were distinct between these two regions. Population differences between Europe and Asian countries were greater than those among China, Korea and Japan, and the *T*. *callipaeda* populations from Europe and Asia should be divided into two separate sub-populations. These two groups started to diverge during the middle Pleistocene.

## Supporting information

S1 Table *Thelazia callipaeda* sampling and data summary for this study. (DOC)

S2 Table Genetic diversity in the *cyt*b, *cox*1, 12S, ITS1, and 18S sequences and the concatenated sequences in the populations of *Thelazia callipaeda* from China. (DOC)

S3 Table Estimated pairwise *F*_ST_ values of sequences between *Thelazia callipaeda* populations. (DOC)

S4 Table Estimated pairwise *F*_ST_ values of *cox*1 sequences between *Thelazia callipaeda* populations from Europe and Asia. (DOC)

S5 Table Mismatch and neutrality tests results of *Thelazia callipaeda* populations from Europe and Asia. (DOC)

S1 Fig Mismatch distribution analyses of Chinese clinical isolates for *cyt*b, *cox*1, 12S, ITS1 and 18S. (DOC)

S2 Fig Alignment of the twenty one *cox*1 haplotypes (Hap1–Hap21) representing *Thelazia callipaeda* from Europe and Asia. (DOC)

S3 Fig Maximum parsimony (MP) and Bayesian phylogenetic trees of *Thelazia callipaeda* from Europe and Asia based on the analysis of the *cox*1 gene. (DOC)
