# Supplementary material for: Population structure analysis of the neglected parasite Thelazia callipaeda revealed high genetic diversity in Eastern Asia isolates
Source: PLoS Negl Trop Dis. 2018 Jan 11;12(1):e0006165. doi: 10.1371/journal.pntd.0006165 (PMC5783425; doi:10.1371/journal.pntd.0006165)
Supplement: S1 Table — (DOC) [file pntd.0006165.s001.doc]

**S1 Table.** *Thelazia callipaeda* sampling and data summary for this study.

| Sample code | Geographical origin | Host origin | Accession number | | | | |
| --- | --- | --- | --- | --- | --- | --- | --- |
|  |  |  | *cox*1 | *cyt*b | 12S | ITS1 | 18S rDNA |
| Europe-h1 | Italy (Piemonte, Basilicata, Calabria); Germany; Netherlands | *Canis familiaris*;  *Vulpes vulpes*;  *Felis catus*;  *Vulpes vulpes*; | AM042549 | N/a | N/a | N/a | N/a |
| SER-B1 | Serbia (Baljevac) | *Canis familiaris* | KJ433982 | N/a | N/a | N/a | N/a |
| SER-B2 |  | *Felis catus* | KJ433983 | N/a | N/a | N/a | N/a |
| ROM-1 | Romania | *Canis familiaris* | KP087796 | N/a | N/a | N/a | N/a |
| ROM-2 |  | *Canis familiaris* | KT716012 | N/a | N/a | N/a | N/a |
| ROM-3 |  | *Canis familiaris* | KT716013 | N/a | N/a | N/a | N/a |
| SLO-TC | Slovakia | *Canis familiaris* | KY476400 | N/a | N/a | N/a | N/a |
| POR-1 | Portugal | *Oryctolagus cuniculus* | KX033489 | N/a | N/a | N/a | N/a |
| HUN-1 | Hungary | *Felis catus* | KX372681 | N/a | N/a | N/a | N/a |
| JAP-O1 | Japan (Okayama) | *Homo sapiens* | AB538283 | N/a | N/a | N/a | N/a |
| JAP-T1 | Japan (Tokyo) | *Canis lupus* | AB852543 | N/a | N/a | N/a | N/a |
| JAP-T2 |  | *Canis lupus* | AB852544 | N/a | N/a | N/a | N/a |
| JAP-T3 |  | *Canis lupus* | AB852545 | N/a | N/a | N/a | N/a |
| JAP-T4 |  | *Canis lupus* | AB852546 | N/a | N/a | N/a | N/a |
| JAP-T5 |  | *Canis lupus* | AB852547 | N/a | N/a | N/a | N/a |
| JAP-T6 |  | *Canis lupus* | AB852548 | N/a | N/a | N/a | N/a |
| JAP-T7 |  | *Canis lupus* | AB852549 | N/a | N/a | N/a | N/a |
| JAP-S1 | Japan (Saitama) | *Canis lupus* | AB852550 | N/a | N/a | N/a | N/a |
| KOR-h8 | Korea (Kou Kuk) | *Canis familiaris* | AM042556 | N/a | N/a | N/a | N/a |
| KOR-h7 |  | *Canis familiaris* | AM042555 | N/a | N/a | N/a | N/a |
| KOR-h6 |  | *Canis familiaris* | AM042554 | N/a | N/a | N/a | N/a |
| CHN-AH-h5 | China (Anhui) | *Canis familiaris* | AM042553 | N/a | N/a | N/a | N/a |
| CHN-AH-h4 |  | *Canis familiaris* | AM042552 | N/a | N/a | N/a | N/a |
| CHN-AH-h3 |  | *Canis familiaris* | AM042551 | N/a | N/a | N/a | N/a |
| CHN-AH-h2 |  | *Canis familiaris* | AM042550 | N/a | N/a | N/a | N/a |
| CHN-HF1 | China (Anhui, Hefei) | *Homo sapiens* | MF795663 | N/a | MF795599 | MF795720 | MF795631 |
| CHN-LA1 | China (Anhui, Liuan) | *Homo sapiens* | MF795664 | N/a | MF795600 | MF795721 | MF795632 |
| CHN-LA2 |  | *Homo sapiens* | MF795665 | N/a | MF795601 | MF795722 | MF795633 |
| CHN-LA3 |  | *Homo sapiens* | MF795666 | N/a | MF795602 | MF795723 | MF795634 |
| CHN-LA4 |  | *Homo sapiens* | MF795667 | N/a | MF795603 | MF795724 | MF795635 |
| CHN-LA5 |  | *Homo sapiens* | MF795668 | N/a | MF795604 | MF795725 | MF795636 |
| CHN-LA6 |  | *Homo sapiens* | MF795669 | N/a | MF795605 | MF795726 | MF795637 |
| CHN-DD1 | China (Liaoning, Dandong) | *Homo sapiens* | MF795670 | MF795695 | MF795606 | MF795727 | MF795638 |
| CHN-DD2 |  | *Homo sapiens* | MF795671 | MF795696 | MF795607 | MF795728 | MF795639 |
| CHN-SL1 | China (Shaanxi, Shangluo) | *Homo sapiens* | MF795672 | MF795697 | MF795608 | MF795729 | MF795640 |
| CHN-SL2 |  | *Homo sapiens* | MF795673 | MF795698 | MF795609 | MF795730 | MF795641 |
| CHN-SL3 |  | *Homo sapiens* | MF795674 | MF795699 | MF795610 | MF795731 | MF795642 |
| CHN-TC1 | China (Shaanxi, Tongchuan) | *Homo sapiens* | MF795675 | MF795700 | MF795611 | MF795732 | MF795643 |
| CHN-TC2 |  | *Homo sapiens* | MF795676 | MF795701 | MF795612 | MF795733 | MF795644 |
| CHN-TC3 |  | *Homo sapiens* | MF795677 | MF795702 | MF795613 | MF795734 | MF795645 |
| CHN-HG1 | China (Hubei, Huanggang) | *Homo sapiens* | MF795678 | MF795703 | MF795614 | MF795735 | MF795646 |
| CHN-HG2 |  | *Homo sapiens* | MF795679 | MF795704 | MF795615 | MF795736 | MF795647 |
| CHN-WH1 | China (Hubei, Wuhan) | *Homo sapiens* | MF795680 | MF795705 | MF795616 | MF795737 | MF795648 |
| CHN-PDS1 | China (Henan, Pingdignshan) | *Homo sapiens* | MF795681 | MF795706 | MF795617 | MF795738 | MF795649 |
| CHN-PDS2 |  | *Homo sapiens* | MF795682 | MF795707 | MF795618 | MF795739 | MF795650 |
| CHN-PDS3 |  | *Homo sapiens* | MF795683 | MF795708 | MF795619 | MF795740 | MF795651 |
| CHN-PDS4 |  | *Homo sapiens* | MF795684 | MF795709 | MF795620 | MF795741 | MF795652 |
| CHN-PDS5 |  | *Homo sapiens* | MF795685 | MF795710 | MF795621 | MF795742 | MF795653 |
| CHN-PDS6 |  | *Homo sapiens* | MF795686 | MF795711 | MF795622 | MF795743 | MF795654 |
| CHN-PDS7 |  | *Homo sapiens* | MF795687 | MF795712 | MF795623 | MF795744 | MF795655 |
| CHN-PDS8 |  | *Homo sapiens* | MF795688 | MF795713 | MF795624 | MF795745 | MF795656 |
| CHN-JZ1 | China (Henan, Jiaozuo) | *Homo sapiens* | MF795689 | MF795714 | MF795625 | MF795746 | MF795657 |
| CHN-JZ2 |  | *Homo sapiens* | MF795690 | MF795715 | MF795626 | MF795747 | MF795658 |
| CHN-LY1 | China (Henan, Luoyang) | *Homo sapiens* | MF795691 | MF795716 | MF795627 | MF795748 | MF795659 |
| CHN-LY2 |  | *Homo sapiens* | MF795692 | MF795717 | MF795628 | MF795749 | MF795660 |
| CHN-ZZ1 | China (Henan, Zhengzhou) | *Homo sapiens* | MF795693 | MF795718 | MF795629 | MF795750 | MF795661 |
| CHN-ZZ2 |  | *Homo sapiens* | MF795694 | MF795719 | MF795630 | MF795751 | MF795662 |
