# Supplementary material for: Population structure analysis of the neglected parasite Thelazia callipaeda revealed high genetic diversity in Eastern Asia isolates
Source: PLoS Negl Trop Dis. 2018 Jan 11;12(1):e0006165. doi: 10.1371/journal.pntd.0006165 (PMC5783425; doi:10.1371/journal.pntd.0006165)
Supplement: S2 Table — (DOC) [file pntd.0006165.s002.doc]

**S2 Table.** Genetic diversity in the *cyt*b, *cox*1, 12S, ITS1, and 18S sequences and the concatenated sequences in the populations of *Thelazia callipaeda* from China. The "SS", "Pi", "Hd" and "S.D." denote sampling size, nucleotide diversity, haplotype diversity and standard deviation, respectively.

| Genes | Population | SS | Haplotypes (Frequencies) | Pi±S.D. | Hd±S.D. |
| --- | --- | --- | --- | --- | --- |
| *cyt*b | DD | 2 | Hap1(2) | 0.00000±0.00000 | 1.000±0.000 |
|  | SL | 3 | Hap2(3) | 0.00000±0.00000 | 1.000±0.000 |
|  | TC | 3 | Hap3(3) | 0.00000±0.00000 | 1.000±0.000 |
|  | HG | 2 | Hap4(2) | 0.00000±0.00000 | 1.000±0.000 |
|  | WH | 1 | Hap5(1) | 0.00000±0.00000 | 1.000±0.000 |
|  | PDS | 8 | Hap6(4), Hap7(4) | 0.00607±0.00100 | 0.571±0.094 |
|  | LY | 2 | Hap6(2) | 0.00000±0.00000 | 1.000±0.000 |
|  | JZ | 2 | Hap8(2) | 0.00000±0.00000 | 1.000±0.000 |
|  | ZZ | 2 | Hap6(1), Hap8(1) | 0.01546±0.00773 | 1.000±0.500 |
|  | Total | 25 |  | 0.01090±0.00096 | 0.873±0.037 |
| *cox*1 | HF | 1 | Hap1(1) | 0.00000±0.00000 | 1.000±0.000 |
|  | LA | 6 | Hap1(6) | 0.00000±0.00000 | 1.000±0.000 |
|  | DD | 2 | Hap2(2) | 0.00000±0.00000 | 1.000±0.000 |
|  | SL | 3 | Hap3(3) | 0.00000±0.00000 | 1.000±0.000 |
|  | TC | 3 | Hap4(3) | 0.00000±0.00000 | 1.000±0.000 |
|  | HG | 2 | Hap5(2) | 0.00000±0.00000 | 1.000±0.000 |
|  | WH | 1 | Hap6(1) | 0.00000±0.00000 | 1.000±0.000 |
|  | PDS | 8 | Hap3(4), Hap7(4) | 0.00087±0.00014 | 0.571±0.094 |
|  | LY | 2 | Hap3(2) | 0.00000±0.00000 | 1.000±0.000 |
|  | JZ | 2 | Hap8(2) | 0.00000±0.00000 | 1.000±0.000 |
|  | ZZ | 2 | Hap3(1), Hap9(1) | 0.00606±0.00303 | 1.000±0.500 |
|  | Total | 32 |  | 0.00599±0.00055 | 0.843±0.040 |
| 12S | HF | 1 | Hap1(1) | 0.00000±0.00000 | 1.000±0.000 |
|  | LA | 6 | Hap1(6) | 0.00000±0.00000 | 1.000±0.000 |
|  | DD | 2 | Hap2(2) | 0.00000±0.00000 | 1.000±0.000 |
|  | SL | 3 | Hap3(3) | 0.00000±0.00000 | 1.000±0.000 |
|  | TC | 3 | Hap4(3) | 0.00000±0.00000 | 1.000±0.000 |
|  | HG | 2 | Hap5(2) | 0.00000±0.00000 | 1.000±0.000 |
|  | WH | 1 | Hap6(1) | 0.00000±0.00000 | 1.000±0.000 |
|  | PDS | 8 | Hap7(4), Hap8(4) | 0.00126±0.00021 | 0.571±0.094 |
|  | LY | 2 | Hap7(2) | 0.00000±0.00000 | 1.000±0.000 |
|  | JZ | 2 | Hap8(2) | 0.00000±0.00000 | 1.000±0.000 |
|  | ZZ | 2 | Hap7(1), Hap8(1) | 0.00221±0.00110 | 1.000±0.500 |
|  | Total | 32 |  | 0.00821±0.00097 | 0.857±0.029 |
| ITS1 | HF | 1 | Hap1(1) | 0.00000±0.00000 | 1.000±0.000 |
|  | LA | 6 | Hap1(6) | 0.00000±0.00000 | 1.000±0.000 |
|  | DD | 2 | Hap2(2) | 0.00000±0.00000 | 1.000±0.000 |
|  | SL | 3 | Hap3(3) | 0.00000±0.00000 | 1.000±0.000 |
|  | TC | 3 | Hap4(3) | 0.00000±0.00000 | 1.000±0.000 |
|  | HG | 2 | Hap5(2) | 0.00000±0.00000 | 1.000±0.000 |
|  | WH | 1 | Hap6(1) | 0.00000±0.00000 | 1.000±0.000 |
|  | PDS | 8 | Hap7(4), Hap8(4) | 0.00222±0.00037 | 0.571±0.094 |
|  | LY | 2 | Hap7(2) | 0.00000±0.00000 | 1.000±0.000 |
|  | JZ | 2 | Hap8(2) | 0.00000±0.00000 | 1.000±0.000 |
|  | ZZ | 2 | Hap7(1), Hap8(1) | 0.00389±0.00194 | 1.000±0.500 |
|  | Total | 32 |  | 0.01002±0.00077 | 0.857±0.029 |
| 18S | HF | 1 | Hap1(1) | 0.00000±0.00000 | 1.000±0.000 |
|  | LA | 6 | Hap2(6) | 0.00000±0.00000 | 1.000±0.000 |
|  | DD | 2 | Hap3(2) | 0.00000±0.00000 | 1.000±0.000 |
|  | SL | 3 | Hap5(3) | 0.00000±0.00000 | 1.000±0.000 |
|  | TC | 3 | Hap4(3) | 0.00000±0.00000 | 1.000±0.000 |
|  | HG | 2 | Hap6(2) | 0.00000±0.00000 | 1.000±0.000 |
|  | WH | 1 | Hap6(1) | 0.00000±0.00000 | 1.000±0.000 |
|  | PDS | 8 | Hap5(4), Hap7(4) | 0.00048±0.00008 | 0.571±0.094 |
|  | LY | 2 | Hap5(2) | 0.00000±0.00000 | 1.000±0.000 |
|  | JZ | 2 | Hap7(2) | 0.00000±0.00000 | 1.000±0.000 |
|  | ZZ | 2 | Hap5(1), Hap7(1) | 0.00083±0.00042 | 1.000±0.500 |
|  | Total | 32 |  | 0.00111±0.00012 | 0.823±0.036 |
