# Supplementary material for: Population structure analysis of the neglected parasite Thelazia callipaeda revealed high genetic diversity in Eastern Asia isolates
Source: PLoS Negl Trop Dis. 2018 Jan 11;12(1):e0006165. doi: 10.1371/journal.pntd.0006165 (PMC5783425; doi:10.1371/journal.pntd.0006165)
Supplement: S3 Table — (DOC) [file pntd.0006165.s003.doc]

**S3 Table.** Estimated pairwise *F*_ST_ values of sequences between *Thelazia callipaeda* populations.

| *cox*1 | HF | LA | DD | SL | TC | HG | WH | PDS | LY | JZ | ZZ |
| --- | --- | --- | --- | --- | --- | --- | --- | --- | --- | --- | --- |
| HF | 0.000 |  |  |  |  |  |  |  |  |  |  |
| LA | 0.000 | 0.000 |  |  |  |  |  |  |  |  |  |
| DD | 1.000 | 1.000* | 0.000 |  |  |  |  |  |  |  |  |
| SL | 1.000 | 1.000* | 1.000 | 0.000 |  |  |  |  |  |  |  |
| TC | 1.000 | 1.000* | 1.000 | 1.000 | 0.000 |  |  |  |  |  |  |
| HG | 1.000 | 1.000* | 1.000 | 1.000 | 1.000 | 0.000 |  |  |  |  |  |
| WH | 1.000 | 1.000 | 1.000 | 1.000 | 1.000* | 1.000 | 0.000 |  |  |  |  |
| PDS | 0.924 | 0.956* | 0.861* | 0.250 | 0.829 | 0.861* | 0.873 | 0.000 |  |  |  |
| LY | 1.000 | 1.000* | 1.000 | 0.000 | 1.000 | 1.000 | 1.000 | 0.158 | 0.000 |  |  |
| JZ | 1.000 | 1.000* | 1.000 | 1.000 | 1.000 | 1.000 | 1.000 | 0.891* | 1.000 | 0.000 |  |
| ZZ | 0.333 | 0.878* | 0.600 | 0.250 | 0.250 | 0.333 | 0.000 | 0.304 | 0.000 | 0.333 | 0.000 |
| 12S | HF | LA | DD | SL | TC | HG | WH | PDS | LY | JZ | ZZ |
| HF | 0.000 |  |  |  |  |  |  |  |  |  |  |
| LA | 0.000 | 0.000 |  |  |  |  |  |  |  |  |  |
| DD | 1.000 | 1.000* | 0.000 |  |  |  |  |  |  |  |  |
| SL | 1.000 | 1.000* | 1.000 | 0.000 |  |  |  |  |  |  |  |
| TC | 1.000 | 1.000* | 1.000 | 1.000 | 0.000 |  |  |  |  |  |  |
| HG | 1.000 | 1.000* | 1.000 | 1.000 | 1.000 | 0.000 |  |  |  |  |  |
| WH | 1.000 | 1.000 | 1.000 | 1.000 | 1.000 | 1.000 | 0.000 |  |  |  |  |
| PDS | 0.873 | 0.926* | 0.686* | 0.876* | 0.921* | 0.948* | 0.940 | 0.000 |  |  |  |
| LY | 1.000 | 1.000* | 1.000 | 1.000 | 1.000 | 1.000 | 1.000 | 0.158 | 0.000 |  |  |
| JZ | 1.000 | 1.000* | 1.000 | 1.000 | 1.000 | 1.000 | 1.000 | 0.158 | 1.000 | 0.000 |  |
| ZZ | 0.778 | 0.962* | 0.667 | 0.903 | 0.939 | 0.947 | 0.895 | -0.455 | 0.000 | 0.000 | 0.000 |
| ITS1 | HF | LA | DD | SL | TC | HG | WH | PDS | LY | JZ | ZZ |
| HF | 0.000 |  |  |  |  |  |  |  |  |  |  |
| LA | 0.000 | 0.000 |  |  |  |  |  |  |  |  |  |
| DD | 1.000 | 1.000* | 0.000 |  |  |  |  |  |  |  |  |
| SL | 1.000 | 1.000* | 1.000 | 0.000 |  |  |  |  |  |  |  |
| TC | 1.000 | 1.000* | 1.000 | 1.000 | 0.000 |  |  |  |  |  |  |
| HG | 1.000 | 1.000* | 1.000 | 1.000 | 1.000 | 0.000 |  |  |  |  |  |
| WH | 1.000 | 1.000 | 1.000 | 1.000 | 1.000 | 1.000 | 0.000 |  |  |  |  |
| PDS | 0.873 | 0.926* | 0.891* | 0.829* | 0.769* | 0.603* | 0.798 | 0.000 |  |  |  |
| LY | 1.000 | 1.000* | 1.000 | 1.000 | 1.000 | 1.000 | 1.000 | 0.158 | 0.000 |  |  |
| JZ | 1.000 | 1.000* | 1.000 | 1.000 | 1.000 | 1.000 | 1.000 | 0.158 | 1.000 | 0.000 |  |
| ZZ | 0.778 | 0.962* | 0.889 | 0.864 | 0.812 | 0.571 | 0.647 | -0.455 | 0.000 | 0.000 | 0.000 |
| 18S | HF | LA | DD | SL | TC | HG | WH | PDS | LY | JZ | ZZ |
| HF | 0.000 |  |  |  |  |  |  |  |  |  |  |
| LA | 0.000 | 0.000 |  |  |  |  |  |  |  |  |  |
| DD | 1.000 | 1.000* | 0.000 |  |  |  |  |  |  |  |  |
| SL | 1.000 | 1.000* | 1.000 | 0.000 |  |  |  |  |  |  |  |
| TC | 1.000 | 1.000* | 1.000 | 1.000 | 0.000 |  |  |  |  |  |  |
| HG | 1.000 | 1.000* | 1.000 | 1.000 | 1.000 | 0.000 |  |  |  |  |  |
| WH | 1.000 | 1.000 | 1.000 | 1.000 | 1.000 | 1.000 | 0.000 |  |  |  |  |
| PDS | 0.619 | 0.783* | 0.686* | 0.250 | 0.721* | 0.807* | 0.771 | 0.000 |  |  |  |
| LY | 1.000 | 1.000* | 1.000 | 1.000 | 1.000 | 1.000 | 1.000 | 0.158 | 0.000 |  |  |
| JZ | 1.000 | 1.000* | 1.000 | 1.000 | 1.000 | 1.000 | 1.000 | 0.158 | 1.000 | 0.000 |  |
| ZZ | 0.333 | 0.878* | 0.667 | 0.250 | 0.769 | 0.800 | 0.600 | -0.455 | 0.000 | 0.000 | 0.000 |
| *cyt*b | DD | SL | TC | HG | WH | PDS | LY | JZ | ZZ |  |  |
| DD | 0.000 |  |  |  |  |  |  |  |  |  |  |
| SL | 1.000 | 0.000 |  |  |  |  |  |  |  |  |  |
| TC | 1.000 | 1.000 | 0.000 |  |  |  |  |  |  |  |  |
| HG | 1.000 | 1.000 | 1.000 | 0.000 |  |  |  |  |  |  |  |
| WH | 1.000 | 1.000 | 1.000 | 1.000 | 0.000 |  |  |  |  |  |  |
| PDS | 0.523* | 0.663* | 0.685* | 0.561* | 0.660 | 0.000 |  |  |  |  |  |
| LY | 1.000 | 1.000 | 1.000 | 1.000 | 1.000 | 0.158 | 0.000 |  |  |  |  |
| JZ | 1.000 | 1.000 | 1.000 | 1.000 | 1.000 | 0.594* | 1.000 | 0.000 |  |  |  |
| ZZ | 0.467 | 0.688 | 0.706 | 0.500 | -0.067 | -0.013 | 0.000 | 0.000 | 0.000 |  |  |

Significance of χ^2^: * *p*-value < 0.05.
