# Supplementary material for: Population structure analysis of the neglected parasite Thelazia callipaeda revealed high genetic diversity in Eastern Asia isolates
Source: PLoS Negl Trop Dis. 2018 Jan 11;12(1):e0006165. doi: 10.1371/journal.pntd.0006165 (PMC5783425; doi:10.1371/journal.pntd.0006165)
Supplement: S4 Table — (DOC) [file pntd.0006165.s004.doc]

**S4 Table.** Estimated pairwise *F*_ST_ values of *cox*1 sequences between *Thelazia callipaeda* populations from Europe and Asia.

| Population | Europe | Korea | Japan | China |
| --- | --- | --- | --- | --- |
| Europe | 0.000 |  |  |  |
| Korea | 0.961** | 0.000 |  |  |
| Japan | 0.866** | 0.444** | 0.000 |  |
| China | 0.827** | 0.275** | 0.430** | 0.000 |

Significance of χ^2^: ** *p*-value < 0.01.
