# Supplementary material for: Population structure analysis of the neglected parasite Thelazia callipaeda revealed high genetic diversity in Eastern Asia isolates
Source: PLoS Negl Trop Dis. 2018 Jan 11;12(1):e0006165. doi: 10.1371/journal.pntd.0006165 (PMC5783425; doi:10.1371/journal.pntd.0006165)
Supplement: S5 Table — (DOC) [file pntd.0006165.s005.doc]

**S5 Table.** Mismatch and neutrality tests results of *Thelazia callipaeda* populations from Europe and Asia.

| Population | Neutrality Tests | | Mismatch | |
| --- | --- | --- | --- | --- |
|  | Fu’s *F*_S_ | Tajima’s *D* | SSD | RI |
| Total | 0.81614 (0.658) | 0.52459 (0.759) | 0.04813 (0.008) | 0.08394(0.001) |

SSD= Sum of Squared deviation, RI = Raggedness index. Number in parentheses is *P* value.
