# Supplementary material for: Population structure analysis of the neglected parasite Thelazia callipaeda revealed high genetic diversity in Eastern Asia isolates
Source: PLoS Negl Trop Dis. 2018 Jan 11;12(1):e0006165. doi: 10.1371/journal.pntd.0006165 (PMC5783425; doi:10.1371/journal.pntd.0006165)
Supplement: S1 Fig — (DOC) [file pntd.0006165.s006.doc]

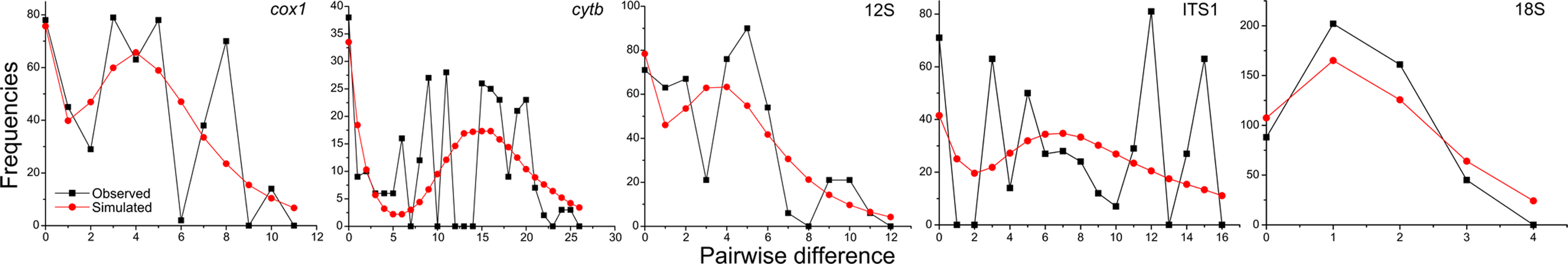


**S1 Figure.** Mismatch distribution analyses of Chinese clinical isolates for *cyt*b, *cox*1, 12S, ITS1 and 18S. The line charts represent the observed frequencies of pairwise differences among haplotypes.
