# Supplementary material for: Population structure analysis of the neglected parasite Thelazia callipaeda revealed high genetic diversity in Eastern Asia isolates
Source: PLoS Negl Trop Dis. 2018 Jan 11;12(1):e0006165. doi: 10.1371/journal.pntd.0006165 (PMC5783425; doi:10.1371/journal.pntd.0006165)
Supplement: S2 Fig — (DOC) [file pntd.0006165.s007.doc]

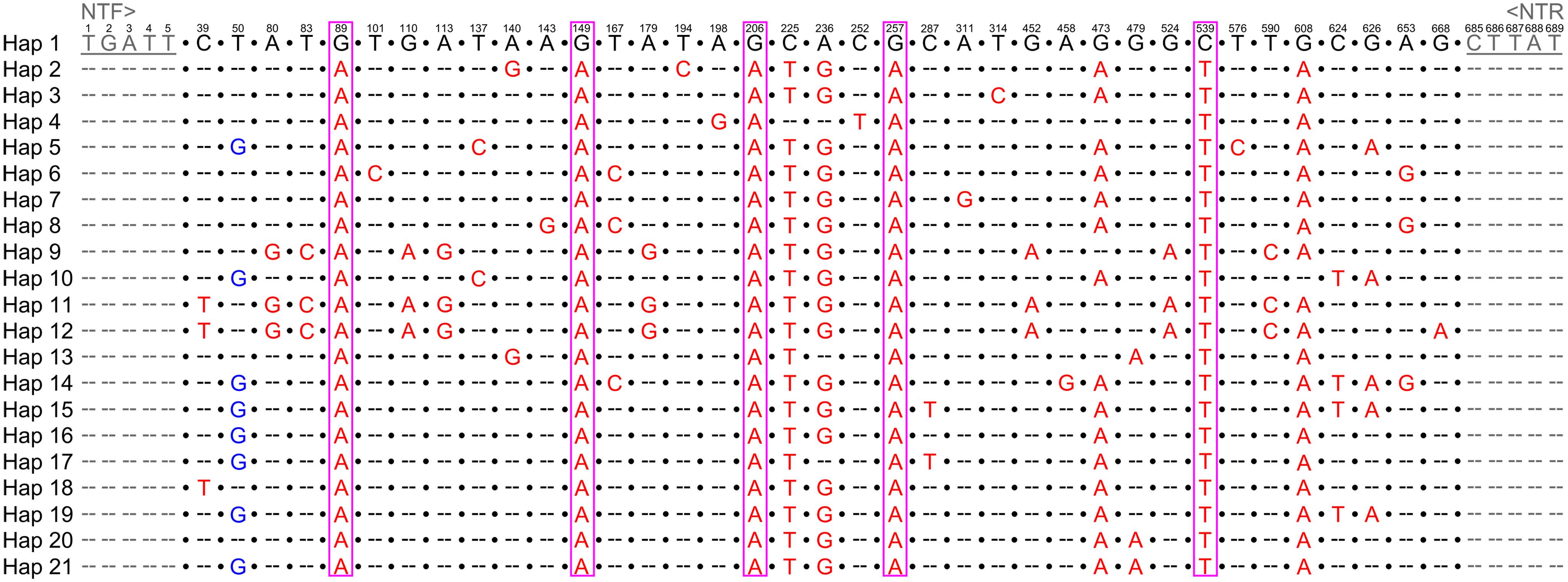


**S2 Figure.** Alignment of the twenty one *cox*1 haplotypes (Hap1–Hap21) representing *Thelazia callipaeda* from Europe and Asia. Identical bases are indicated by a dash line. The number above each base indicates the alignment position of *cox*1. Purple rectangle regions indicate nucleotides which differ consistently between Hap1 from Europe and haplotypes Hap2–Hap21 from Asia. Grey and underlined are the first five bases of the oligonucleotide primers used in the PCR.
