# Supplementary material for: Population structure analysis of the neglected parasite Thelazia callipaeda revealed high genetic diversity in Eastern Asia isolates
Source: PLoS Negl Trop Dis. 2018 Jan 11;12(1):e0006165. doi: 10.1371/journal.pntd.0006165 (PMC5783425; doi:10.1371/journal.pntd.0006165)
Supplement: S3 Fig — (DOC) [file pntd.0006165.s008.doc]

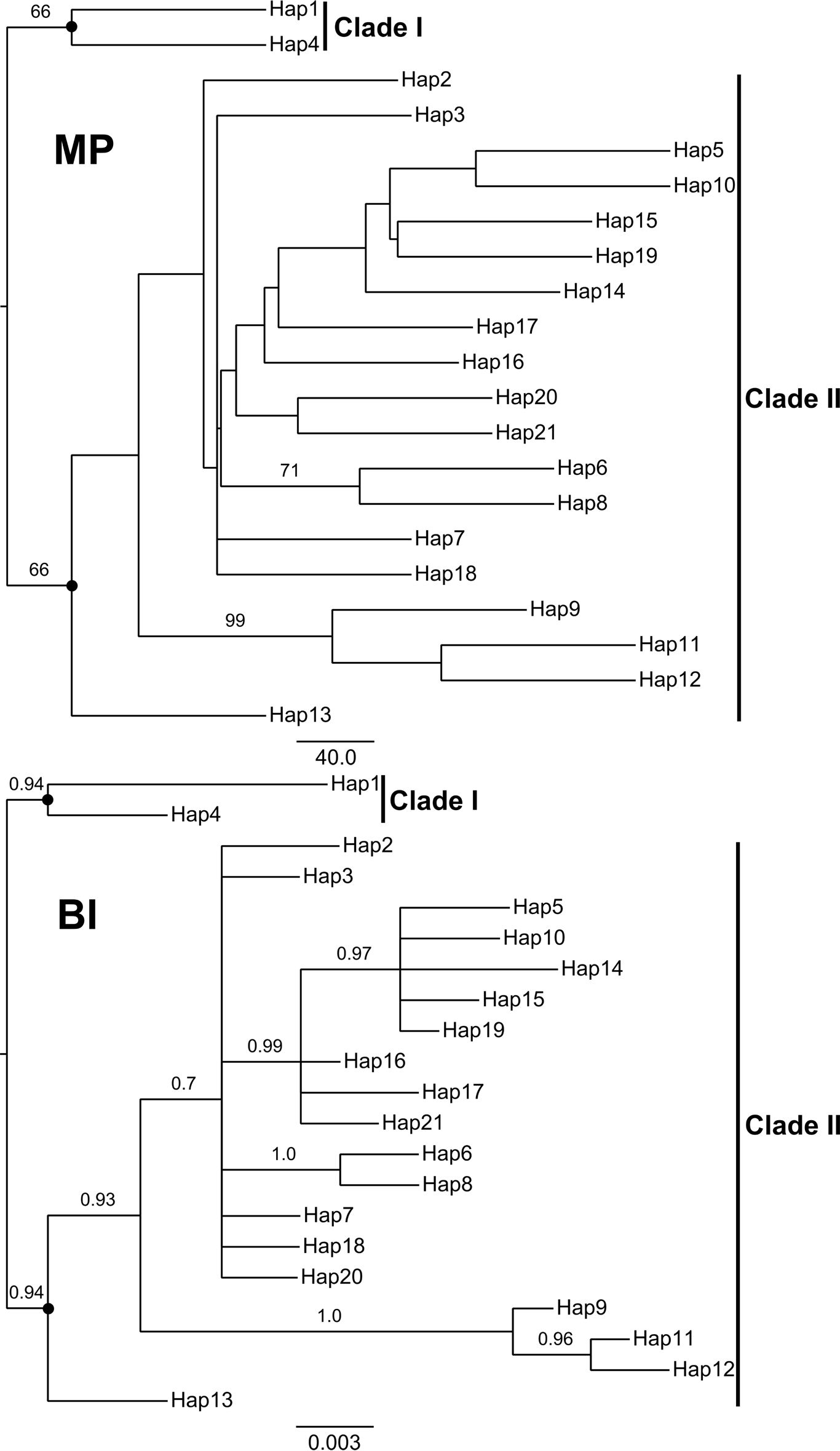


**S3 Figure.** Maximum parsimony (MP) and Bayesian phylogenetic trees of *Thelazia callipaeda* from Europe and Asia based on the analysis of *cox*1 gene. Only bootstrap values above 60 and posterior probabilities values above 0.6 are shown.
